# Clinical phenotype and diagnosis of arrhythmogenic right ventricular cardiomyopathy in pediatric patients carrying desmosomal gene mutations

**DOI:** 10.1016/j.hrthm.2011.06.026

**Published:** 2011-11

**Authors:** Barbara Bauce, Alessandra Rampazzo, Cristina Basso, Elisa Mazzotti, Ilaria Rigato, Alexandros Steriotis, Giorgia Beffagna, Alessandra Lorenzon, Marzia De Bortoli, Kalliopi Pilichou, Martina Perazzolo Marra, Francesco Corbetti, Luciano Daliento, Sabino Iliceto, Domenico Corrado, Gaetano Thiene, Andrea Nava

**Affiliations:** ⁎ Department of Cardiac, Thoracic and Vascular Sciences, University of Padua, Padua, Italy; † Department of Biology, University of Padua, Padua, Italy; ‡ Department of Medical Diagnostic Sciences and Special Therapies, University of Padua, Padua, Italy; § Division of Radiology, Padua Hospital, Padua, Italy

**Keywords:** ARVC, arrhythmogenic right ventricular cardiomyopathy, BSA, body surface area, CMR, cardiac magnetic resonance, ECG, electrocardiogram, LE, late enhancement, LV, left ventricular, ventricle, LVEF, left ventricular ejection fraction, LVEDV, left ventricular end diastolic volume, MM, multiple mutations, PLAX, parasternal long axis, PSAX, parasternal short axis, PVC, premature ventricular complex, RV, right ventricular, RVEDA, right ventricular end-diastolic area, RVEDV, right ventricular end-diastolic volume, RVFS, right ventricular fraction shortening, RVOT, right ventricular outflow tract, SAECG, signal-averaged electrocardiogram, VF, ventricular fibrillation, VT, ventricular tachycardia

## Abstract

**Background:**

Arrhythmogenic right ventricular cardiomyopathy (ARVC) is an inherited heart muscle disease carrying a risk of sudden death. Information about the clinical features during childhood and the age at disease onset is scanty.

**Objective:**

The aim of the study was to describe the ARVC phenotype as its initial clinical manifestation in a pediatric population (<18 years) with desmosomal gene mutations.

**Methods:**

Fifty-three ARVC desmosomal gene mutation carriers (mean age 12.3 ± 3.9 years) were investigated by electrocardiogram (ECG), signal-averaged ECG, 24-hour Holter, echocardiogram, and contrast-enhanced cardiac magnetic resonance (CMR).

**Results:**

None of the children ≤10 years old fulfilled the 1994 criteria, as opposed to six (33%) aged 11–14 years and eight aged >14 years (42%). At the end of follow-up (9 ± 7 years), 21 (40%) fulfilled the 1994 diagnostic criteria (mean age 16 ± 4 years). By using the 2010 criteria in subjects aged ≤18 years, 53% were unaffected, versus 62% by using the traditional criteria. More than two-thirds of affected subjects had moderate-severe forms of the disease. Contrast-enhanced CMR was performed in 21 (40%); of 13 unaffected gene mutation carriers, six showed ARVC morphological and/or tissue abnormalities.

**Conclusion:**

In pediatric ARVC mutation carriers, a diagnosis was achieved in 40% of cases, confirming that the disease usually develops during adolescence and young adulthood. The 2010 modified criteria seem to be more sensitive than the 1994 ones in identifying familial pediatric cases. Contrast-enhanced CMR can provide diagnostic information on gene mutation carriers not fulfilling either traditional or modified criteria. Management of asymptomatic gene mutation carriers remains the main clinical challenge.

## Introduction

Major advances have been made in our understanding of arrhythmogenic right ventricular cardiomyopathy (ARVC) in the last few years, primarily involving the detection of several disease-causing genes that encode mainly for desmosomal proteins.^1–9^ Genotype-phenotype correlation studies have demonstrated that subjects carrying mutations of the same gene may have different clinical features and disease extent, in terms of right (RV) and left (LV) ventricular dimensional and kinetics as well as of electrical instability.^10–15^ Most available data on the clinical picture of ARVC come from observations in adults, while there is little information on the signs and symptoms of the disease in childhood and adolescence^16–18^ and none on genotype-phenotype correlation in this age group.

The present study was designed to analyze a series of ARVC-desmosomal gene mutation carriers first assessed at a young age in an outpatient clinic as part of a family screening program for inherited cardiomyopathies to elucidate the disease phenotype in terms of clinical onset and natural history.

## Methods

### Study population

Fifty-three subjects (31 males and 22 females) belonging to 27 families and evaluated at our center from 1980 to 2008 before the age of 18 and carrying desmosomal genes mutations were analyzed. The study was partly retrospective because genetic data were obtained for some cases when they had already joined the yearly follow-up program after family screening for ARVC. Based on their age at first observation, patients were divided into three age groups: group A ≤10 years; group B 11–14 years; group C >14–18 years.

## Genetic study

Screening for mutations in the plakophilin-2 (*PKP2*), desmoplakin (*DSP*), desmoglein-2 (*DSG2*), desmocollin-2 (*DSC2*), and plakoglobin (*JUP*) genes was done by denaturing high-performance liquid chromatography and direct sequencing. The analysis was conducted using the WAVE Nucleic Acid Fragment Analysis System 3500HT with DNASep HT cartridge technology (Transgenomic Ltd NE, USA). All amplimers showing abnormal elution profiles were directly sequenced using the BIG DYE dideoxy-terminator chemistry (Perkin Elmer, USA) on an ABI 377 DNA sequencer (PE Applied Biosystems, USA). Chromas 1.5 software (Technelysium, USA) and LASERGENE package computer programmes (DNASTAR, USA) were used to edit, assemble, and translate sequences. A control group of 250 healthy, unrelated subjects (500 alleles) drawn from the Italian population was used to rule out the possibility of DNA polymorphisms. For ethical reasons, in asymptomatic minors, genetic screening was performed only after detection of the mutation in the family and after parents' written consent as recommended by the European Society of Human Genetics.^19^

## Cardiological screening

Subjects were assessed for the first time because they had a family history of ARVC (n = 24) or of juvenile sudden death (n = 16) due to arrhythmic symptoms (n = 9), chest pain episodes (n = 3), and electrocardiogram (ECG) abnormalities (n = 1). All subjects with clinical signs of the disease entered a follow-up program with visits every 6 months; otherwise, they had yearly visits.

The study protocol included 12-lead ECG, signal-averaged ECG (SAECG), 24-hour Holter, and two-dimensional echocardiogram, as described elsewhere.^16^ Cardiac magnetic resonance (CMR) with gadolinium as contrast agent was performed in selected cases. The following clinical parameters were considered: age at time of diagnosis and at enrollment for the study, family history of ARVC and/or of juvenile sudden death, and clinical presentation. Any palpitations, syncope, sudden death or aborted sudden death, and chest pain were carefully investigated.

The ECG parameters analyzed were incomplete or complete right bundle branch block, negative T waves on precordial or limb leads, epsilon waves, low QRS voltages, and ST segment elevation in precordial leads. As far as the presence of negative T waves in V1–V3, we did not consider these ECG features among the diagnostic criteria in subjects <12 years old when using 1994 criteria^20^ and <14 years old when using the modified diagnostic criteria.^21^

SAECG was performed with the MAC 15 system (Marquette Inc.). Time-domain analysis was done using three different filters (25, 40, 80 Hz) and was considered positive when at least two parameters were abnormal with two filter setting.^22^ When using 2010 criteria, SAECG was considered positive if late potentials were present in one or more of three parameters in the absence of a QRS duration of ≥110 ms.^21^ The presence of premature ventricular complexes (PVCs) was considered as a criterion, with the thresholds fixed by the two task forces diagnostic criteria.^20,21^

The echocardiogram was done with a 2.5- to 4-MHz transducer (HP 5500) with M-Mode, two-dimensional, and Doppler examination. LV volumes were calculated using an ellipsoid biplane area-length model derived from LV images in the apical four-chamber view. RV volumes were calculated using an area-length method derived from orthogonal planes (apical four-chamber and short-axis subcostal views). RV fractional area change was measured from the apical four-chamber view. Moreover, RV dimensions that have been proposed by the updated 2010 criteria were also considered.^21^ Parasternal long axis (PLAX) right ventricular outflow tract (RVOT) was calculated from the anterior RV wall to the RV septum in the PLAX, and parasternal short axis (PSAX) RVOT was obtained in PSAX view as the maximum distance between the anterior aortic wall and the RV free wall. Measures were corrected for body size area (BSA).

CMR was performed on a 1.0-T clinical scanner (Harmony, Siemens, Germany) using a phased-array cardiac receiver coil. After the intravenous administration of gadolinium chelate (0.2 mmol/kg), inversion recovery prepared breath-hold cine gradient-echo images were obtained^23^ to assess late enhancement (LE) according to a previously described method.^24,25^ CMR was not performed in asymptomatic subjects less than 10 years of age for ethical reasons. In the other cases, CMR was performed in subjects with no contraindications and with the parents' written consent.

ARVC was diagnosed according to the 1994 task force criteria.^20^ The subjects affected were classified as having mild, moderate, or severe disease according to the RV end-diastolic volume (RVEDV) and the presence of RV wall motion abnormalities.^16^ The recently proposed modified criteria were applied,^21^ classifying subjects as affected, borderline, or unaffected. Finally, the LE on CMR was assessed in family members as a potential new diagnostic criterion.

## Statistical analysis

Data are expressed as mean value ± standard deviation for continuous variables and as frequencies with percentages for categorical variables. All continuous variables are expressed as the mean ± standard deviation. An unpaired Student's *t*-test was used to compare normally distributed data. Lifelong event-free survival rates were estimated using the Kaplan-Meier method, and comparisons were drawn using the log-rank test. The following major events were considered: sustained ventricular tachycardia (VT), ventricular fibrillation (VF), cardiac arrest, heart failure, syncope, and chest pain. The *P*-values were two-sided and considered statistically significant when <.05. Data were analyzed by SPSS 18 for Windows (SPSS Inc., Chicago).

## Results

Among the 53 subjects evaluated under 18 years of age, 26 (49%) had a gene mutation in *DSP*, 12 (23%) in *PKP2*, six (11%) in *DSG2*, and nine (17%) had multiple mutations (MM) in one or two different genes (Table 1, with more detailed information in Supplemental Table 1).^4,7,10,13,15,26–40^ None of the mutations were found in the control population.

## Clinical phenotype at first evaluation

None of the 16 subjects belonging to group A (six males, 10 females, mean age 7.2 ± 2.1 years) fulfilled the 1994 ARVC diagnostic criteria. They were all assessed owing to a family history of ARVC, except for one who had chest pain with myocardial enzyme release without ECG changes in the presence of angiographically normal coronary arteries.

Among the 18 patients belonging to group B (15 males, three females, mean age 12.6 ± 1 years), 12 (67%) were studied owing to a family history of ARVC, two for chest pain, three for ventricular arrhythmias, and one because of abnormal ECG findings. Six patients (33%, five males, one female, age range 11–14 years) were diagnosed with ARVC, and they all had ventricular arrhythmias (sustained VT in one, nonsustained VT in three, isolated PVCs in two). Two had a mild form of the disease, two a moderate form, and two a severe form.

Among the 19 subjects belonging to group C (11 males, eight females, mean age 16.3 ± 1 years), ARVC diagnosis was made in eight (42%, six males, two females, age range 15–18 years). The majority of subjects (n = 13, 68%) were studied owing to a family history of ARVC, and the other six (32%) were studied for arrhythmic symptoms. All the affected patients had ventricular arrhythmias (sustained VT in four, nonsustained VT in two, PVCs in two). Two-dimensional echocardiogram demonstrated severe disease in two, moderate in five, and mild in one.

Thus, a total of 14 subjects (26%, 11 males and three females, mean age 15 ± 2 years) were diagnosed with ARVC at first evaluation, whereas the majority (39 [74%], 20 males and 19 females, mean age 11 ± 4 years) showed no signs or symptoms of the disease.

## Clinical phenotype of index cases and family members

Among 53 subjects, 40 were family members. Among these, an ARVC diagnosis using modified criteria was obtained in nine cases (22.5%) at the age of 17.8 ± 5.1 years. Ventricular arrhythmias were present in 27% of family members versus 100% of index cases (*P* = .00001), ECG was abnormal in 15% of family members versus 92% of index cases (*P* = .00001), and SAECG was positive in 15% of family members versus 77% of index cases (*P* = .00001). Moreover, RV dimensions evaluated with RV PLAX/BSA and RV PSAX/BSA were found to be significantly lower in family members compared with index cases.

## Twelve-lead ECG

The ECG was found to be abnormal in 12 (23%) patients at first evaluation, and in 18 (34%) at last follow-up. ECG findings are given in Table 2.

## SAECG

Late potentials were detected in 12 patients (23%) at first evaluation (none in group A, six in group B, and six in group C) and in another eight patients during follow-up (two in group A, five in group B, and one in group C).

## Two-dimensional echocardiogram

Twelve subjects (23%) had typical ARVC features at their first assessment (none in group A, six in group B, and six in group C), which appeared in another six during the follow-up (two in group A, two in group B, and two in group C). Disease progression in terms of morphological abnormalities was demonstrated during follow-up in 12 cases (two in group A, three in group B, and seven in group C).

## Ventricular arrhythmias

At first evaluation, 17 subjects (32%) showed ventricular arrhythmias (one in group A, eight in group B, and eight in group C). In particular, a sustained VT was detected in five subjects (one in group B and four in group C), and nonsustained ventricular arrhythmias in the other 12 (one in group A, seven in group B, and four in group C). A detailed analysis of PVC morphology on 12-lead ECG was available in nine subjects, showing a left bundle branch block morphology in all (with inferior axis in five and superior axis in four).

## Clinical features in relation to genetic results

At first evaluation, ARVC was diagnosed in three of 26 *DSP* mutation carriers (12%; two males and one female, age 15 ± 2 years); in five of twelve *PKP2* carriers (42%; four males and one female, mean age 14 ± 1.6 years); in one of six *DSG2* carriers (17%; male, age 11 years); and in five of nine MM carriers (56%; four males and one female, mean age 16 ± 1.5 years; Table 3). By comparing the clinical and instrumental findings in patients carrying different ARVC gene mutations, no statistically significant differences among gene carriers appeared (Table 4). During the follow-up, *DSP* mutation carriers had a greater disease progression, in terms of RV-LV dilatation and dysfunction as well as of SAECG findings, even if they showed smaller RV dimensions at first evaluation (Table 5). At the end of follow-up among the 20 subjects who achieved the diagnosis, seven belonged to the *DSP* group (27% of all *DSP* carriers), six to the *PKP2* group (50% of all PKP2 carriers), one to the *DSG2* group (17% of all DSG2 carriers), and six to the *MM* group (67% of all *MM* carriers).

## CMR

CMR was performed in 21 (40%) out of 53 subjects and revealed morphofunctional abnormalities of the RV in nine (43%) and LV in four (19%). Contrast-enhanced CMR showed LE in 12 subjects (57%), which was confined to the RV in two and to the LV in five and was biventricular in five (see Table 6).

**Figure 1:**
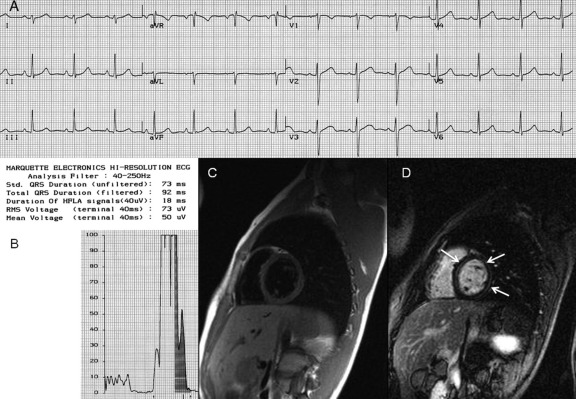
**A:** 12-lead ECG of a 10-year-old family member (DSP mutation carrier) who did not fulfill the 1994 and recently modified diagnostic criteria. The ECG trace is normal. **B:** SAECG of the same patient, with no late potentials at 40 filter settings. **C:** CMR of the same patient with T1 sequence showing no fat infiltration. **D:** CMR of the same patient showing LE on the LV wall (*arrows*).

In eight gene mutation carriers (all males, age range 17–30 years, mean 21 ± 6 years), CMR confirmed an already established diagnosis of ARVC. Among the remaining 13 who did not fulfill either the 1994 or the 2010 diagnostic criteria (eight males, five females, age range 10–26 years, mean 17 ± 6 years), contrast-enhanced CMR detected LE in six (46%), that is, five in the LV and one in the anterior RV free wall. With regard to the eight patients not fulfilling the criteria who underwent a CMR examination before the age of 18, five showed LV LE and one RV LE in the setting of normal RV and LV dimensions and kinetic features (Figure 1).

## ARVC diagnosis in the pediatric age (≤18 years)

By applying the 1994 criteria,^20^ ARVC was diagnosed in 20 out of 53 desmosomal gene mutation carriers (38%, 15 males and five females, age range 11–18 years, mean 15 ± 2 years), while the other 33 (62%) were considered unaffected. Using the recently modified diagnostic criteria,^21^ 21 cases subjects were labeled as affected (40%, age range 11–18 years, 16 males and five females, mean age at diagnosis 15 ± 2 years), four as borderline (8%), and 28 as unaffected (53%). Contrast-enhanced CMR identified LE in an additional five cases by the age of 18 years.

## Therapy

Antiarrhythmic drug therapy was performed in 17 patients (beta-blocker in two, sotalol in 10, propaphenon in one, amiodaron + beta-blocker in two, and dysopiramide + beta-blocker in two). Moreover, a total of four patients received an implantable cardioverter-defibrillator (three males, one female, age range 17–30).

## Follow-up

During 9 ± 7 years of follow-up, seven gene mutation carriers previously considered unaffected were diagnosed as affected with ARVC (four males and three females, age range 15–30 years, mean 18 ± 5 years); three of them had a moderate form and four had a mild form of the disease. Thus, at the end of follow-up a total of 21 subjects (40%, mean age 16 ± 4 years) fulfilled the 1994 diagnostic criteria. By applying recently modified criteria, one additional subject was diagnosed, for a total of 22 affected cases (41.5%), five borderline (9.5%), and 26 unaffected (49%). Nine subjects (seven males and two females), already considered to be affected at first evaluation, showed disease progression in terms of ECG, SAECG, two-dimensional echo abnormalities, and electrical instability.

**Figure 2:**
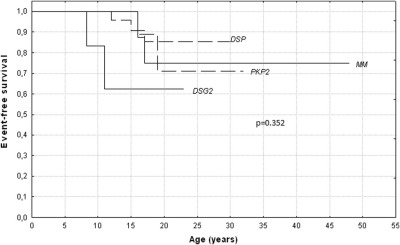
Event-free survival rates in terms of major events according to the underlying genetic background.

With regard to ventricular arrhythmias, during follow-up, one patient (female, 20 years old) experienced a sustained VT and two a VF. Of these, one who had been assessed at the age of 13 and considered unaffected according to both the 1994 and the modified criteria died suddenly when he was 15 years old as first clinical manifestation of the disease.^10^ The other was affected by a moderate form of the disease with isolated PVCs (<1,000/24 hour) and at the age of 18 he had a cardiac arrest and was successfully resuscitated. In addition, two subjects who had previously experienced a VT episode had a VT recurrence (at the age of 17 and 30 years). None of the patients developed heart failure. Finally, lifelong event-free survival rates estimated using the Kaplan-Meier method did not reveal significant differences in terms of major events between patients carrying mutations of different desmosomal genes (*P* = .352; Figure 2).

## Discussion

ARVC is an inherited heart muscle disease characterized by progressive myocardial loss with fibrofatty replacement. Previous studies have shown that, although it is genetically determined, the disease is not present at birth, and it is usually diagnosed in young adulthood when the clinical signs appear, cardiac arrest sometimes being the presenting manifestation.^1,2,16,41^ By analyzing a series of subjects carrying ARVC-related desmosomal gene mutations who were evaluated for the first time before the age of 18 years as part of a family screening program for inherited cardiomyopathies and followed up over a substantial period of time, we gained some new insights into the ARVC phenotype and clinical onset.

### Age at disease onset

The detection of diagnostic criteria in 38% of subjects younger than 18 years old suggests that the cardiac phenotype appears in young adolescence in a significant number of subjects carrying ARVC-related gene mutations, while its clinical onset beyond the age of 18 is less common. In our series, the age at diagnosis using both the 1994^20^ and the modified 2010 diagnostic criteria^21^ was comparable to that reported in other pediatric populations.^17,18^

Moreover, CMR provided additional diagnostic information, since six of the eight subjects <18 years old who did not fulfill the diagnostic criteria revealed LE, localized mainly to the LV, with no other morphological or kinetic abnormalities detectable by echo. This CMR feature has already been reported in ARVC patients^35,42,43^ and supports the hypothesis that the pathologic hallmark of the disease, that is, the myocardial loss followed by fibrofatty replacement, should not be considered exclusive to the RV because it is often biventricular or confined to the LV.^2^

### Clinical phenotype in young subjects

ARVC can be characterized by a severe phenotype from an early age, as demonstrated by our pediatric series. As far as the arrhythmic profile, sustained ventricular arrhythmias were found at first evaluation in nearly one-third of our gene mutation carriers, and two subjects suffered a sudden death and an aborted sudden death. These data confirm the concept that in young patients the risk of life-threatening ventricular arrhythmias is high, probably owing to the peculiar stage of the pathologic process, characterized by an abrupt myocardial loss, followed by fibrofatty substitution, to mimic acute myocardial infarction.^10^ It is worth adding that in a transgenic mouse model of *DSG2*-related ARVC, myocyte necrosis was recently found to be the key initiator of progressive myocardial dystrophy and that this did not occur at birth.^44^

### Genotype-phenotype correlations

Subjects carrying *DSP* mutations had a worse disease progression in terms of RV and LV dilatation and SAECG features during follow-up, even though *DSP* carriers at first evaluation showed smaller RV dimensions. However, event-free survival rates did not identify any prognostic role of the genetic background in terms of major events. Prospective studies on larger series of genotyped ARVC patients are needed to confirm and expand these preliminary data.

### ARVC diagnostic criteria

According to the new criteria, since a causative mutation is a major criterion, you need only some additional clinical features to reach the diagnosis. In fact, the recently modified criteria^21^ were more sensitive than the 1994 ones in identifying familial pediatric cases by reducing the prevalence of unaffected individuals from 62% to 53% under the age of 18. Moreover, contrast-enhanced CMR could detect the presence of LE, mainly localized to the LV, even in the absence of wall thinning or kinetic abnormalities in a substantial proportion of apparently “healthy” carriers. This supports the concept that genetic data need to be considered among the ARVC diagnostic criteria, as recommended in the recently modified 2010 criteria, and that contrast-enhanced CMR should enter the diagnostic work-up at an early stage.

### Clinical management of desmosomal gene mutations carriers

While identifying a desmosomal gene mutation in a person already diagnosed with ARVC does not substantially influence the medical approach to the patient, genotyping the clinically unaffected family members raises the issue of how to manage these so-called healthy carriers. In our opinion, competitive sport should be forbidden and CMR should be repeated yearly because gene mutation carriers can develop the clinical signs of the disease during adolescence and young adulthood, with life-threatening ventricular arrhythmias occurring within a relatively short period of time. Patients should also be advised to report any symptoms such as syncope, chest pain, asthenia, or palpitations promptly. Although the issue of eligibility for competitive sports remains debatable for gene mutation carriers with no clinical signs of the disease (and even with negative CMR), we believe that strenuous effort should be avoided.^45^

### Limitation

To define disease extent, we classified affected subjects as having a mild, moderate, or severe disease, according to the RVEDV and the presence of RV wall motion abnormalities, as we did elsewhere.^16^ However, we recognize that this subclassification into mild, moderate, and severe forms is not widely accepted, particularly when calculated on echocardiographically derived RV volumes. For these reasons, two-dimensional echo RV dimensions that have been recently proposed by the updated 2010 criteria were also used.

### Future aims of the research

The evidence of a different disease penetrance in subjects carrying the same gene mutations supports the hypothesis that additional factors, both exogenous and endogenous, may play a role in modulating the ARVC phenotype. These factors may include athletic activity and male gender, since competitive athletes seem to be exposed to the risk of an earlier onset and more rapid progression of the disease phenotype^45^ and women appear to be affected less frequently than men, although they may have severe forms of the disease.^46^ A better understanding of the additional modulating factors will contribute to the early identification of gene mutation carriers at higher risk to develop an earlier and more severe clinical picture, with the aim to adopt timely lifestyle and therapeutic preventive strategies.

## Figures and Tables

**Table 1:** Mutations in desmosomal genes involved in ARVC

Gene and exon	Nucleotide change	Amino acid change	n	References
*PKP2:*				
Ex1	c.83delC	p.S29AfsX10	1	
Ex1	c.184_185delCA	p.Q62DfsX23	1	26
Ex2	c.227A→G	p.N76S	2	26
Ex7	c.1643delG	p.G548VfsX15	1^a^	15, 27–32
Ex10	c.2013delC	p.K672RfsX12	6	15, 26, 28, 30, 31
Ex12	c.2333T→C	p.I778T	1	29
*DSP:*				
Ex1	c.88G→A	p.V30M	7	29, 33, 34
Intr2	c.273+5G→A	/	2	26
Intr3	c.423−1G→A	/	1	10
Ex7	c.897C→G	p.S299R	6	4, 10
Ex11	c.1372A→T	p.N458Y	1	26
Ex23	c.3337C→T	p.R1113X	2	26, 35
Ex23	c.3774C→A	p.D1258E	1	26
Ex23	c.4803G→A	p.M1601I	2	26
Ex23	c.4961T→C	p.L1654P	1	29, 33
Ex23	c.5324G→T	p.R1775I	1	10
Ex24	c.7622G→A	p.R2541K	1	33
3'UTR	c.⁎9T→A	/	1	
*DSG2*				
Ex4	c.298G→C	p.G100R	1	7, 36
Ex9	c.1174G→A	p.V392I	2	29, 33, 37–39
Ex12	c.1672C→T	p.Q558X	2	7, 36
Ex15	c.2990delG	p.G997VfsX20	1	26
Multiple mutations:				
*PKP2* ex1	c.148_151delACAG	T50SfsX61	1	13, 27, 29, 33, 39
*DSG2* ex9	c.1174G→A	p.V392I	29, 33, 37, 38, 39
*PKP2* ex10	c.2119C→T	p.Q707X	2	29, 33, 36
*DSP* ex23	c.4961T→C	p.L1654P	29, 33
*DSP* ex9	c.1124A→T	p.N375I	1	40
*DSG2* ex13	c.1912G→A	p.G638R	40
*DSP* ex11	c.1408A→G	p.K470E	1	36
*DSP* ex13	c.1696G→A	p.A566T	36
*DSP* ex23	c.3203_3204delAG	p.E1068VfsX19	2	26
*DSG2* ex15	c.2773C→T	p.P925S	26
*DSP* ex23	c.5324G→T	p.R1775I	1	10
*DSG2* ex6	c.689A→G	p.E230G	26
*DSG2* ex9	c.1254_1257insATGA	p.D419X	1	7, 36
*DSG2* ex15	c.2990delG	p.G997VfsX20	26

a Mutation c.

⁎ 495C>T in the *TGFbeta3* gene was also detected in this patient.

**Table 2:** ECG features of 53 desmosomal gene mutation carriers at first and last evaluation

	Abnormal ECG (first/last evaluation)	RV conduction delay (first/last evaluation)	Negative T wave V1–V3 (first/last evaluation)	Negative T wave >V3 (first/last evaluation)	Low QRS voltages (first/last evaluation)	Q wave inferior leads (first/last evaluation)	Epsilon wave (first/last evaluation)	ST segment elevation V1–V2 (first/last evaluation)
Group A (n = 16)	0/2 (12)	1 (6)/0	5 (31)/3 (19)	0/0	0/1 (6)	0/0	0/0	0/0
Group B (n = 18)	8 (44)/10 (55)	5 (28)/5 (28)	5 (28)/4 (22)	1 (6)/4 (22)	1 (6)/2 (11)	0/0	1 (6)/2 (11)	2 (11)/2 (11)
Group C (n = 19)	6 (32)/8(41)	3 (16)/5 (26)	4 (21)/4 (21)	2 (11)/2 (11)	2 (11)/3 (16)	1 (5)/1 (5)	0/0	0/0

*Note:* RV: right ventricular. Numbers in parentheses are percents.

**Table 3:** ARVC diagnosis in relation to gene mutation

	DSP (n = 26)	PKP2 (n = 12)	DSG2 (n = 6)	MM (n = 9)
Index cases	4 (15)	4 (33)	1 (17)	4 (44)
Diagnosis at first evaluation^a^	3 (12)	5 (42)	1 (17)	5 (56)
Diagnosis at follow-up^a^ <18 years	8 (31)	5 (42)	1 (17)	6 (67)
Diagnosis at last follow-up^a^	8 (31)	6 (50)	1 (17)	6 (67)
Diagnosis at first evaluation considering proposed modified criteria^b^	3 (12)	5 (42)	1 (17)	5 (56)
Diagnosis at follow-up < 18 years considering proposed modified criteria^b^	8 (31)	5 (42)	1 (17)	7 (70)
Diagnosis at last follow-up considering proposed modified criteria^b^	8 (31)	6 (50)	1 (17)	7 (78)
Age at diagnosis^a^ (<18 years)	16 ± 2	17 ± 7	11	16.5 ± 1
Age at diagnosis^b^ (<18 years) considering proposed modified criteria	16 ± 2	17 ± 7	11	17 ± 1
Disease extent at diagnosis^a^ (<18 years):				
Mild	3	2	0	1
Moderate	3	2	0	3
Severe	2	2	1	3
Ventricular arrhythmias (<18 years)	11 (42)	6 (50)	2 (33)	7 (78)

Note: Numbers in parentheses are percents.

a Diagnosis made using the 1994 task force criteria.^20^

b Diagnosis made using the 2010 modified criteria.^21^

**Table 4:** SAECG and two-dimensional echo features at first evaluation in 53 desmosomal genes mutation carriers

Gene mutations	No.	Age of patients	RVOT (PLAX)/BSA	RVOT (PSAX)/BSA	RVEDA, cm^2^	RVFS	RV kinetic abn, n (%)	LVEDV, mL/m^2^	LVEF, %	LV kinetic abn, n (%)	Positive SAECG, n (%)
DSP	26	12 ± 3.94	12.72 ± 2.94	16.39 ± 3.54	15 ± 4	37 ± 8	3 (12)	56 ± 8	63 ± 5	1 (4)	3 (12)
PKP2	12	13 ± 3.7	20.05 ± 4.72	23.11 ± 8.3	21 ± 6	36 ± 7	4 (33)	61 ± 9	62 ± 5	0	3 (40)
DSG2	6	10.8 ± 3.6	18.32 ± 6.46	20.36 ± 6.72	17 ± 6	37 ± 6	1 (17)	58 ± 10	61 ± 3	0	1 (17)
MM	9	13 ± 4.9	21.71 ± 9.44	22.68 ± 9.15	20 ± 7	39 ± 12	5 (56)	56 ± 13	62 ± 8	2 (22)	5 (56)
DSP vs. PKP2		0.460	0.00001	0.001	0.0008	0.712	0.131	0.093	0.570	0.730	0.055
DSP vs. DSG2		0.527	0.0026	0.047	0.305	1	0.744	0.594	0.332	0.622	0.744
PKP2 vs. DSG2		0.247	0.525	0.493	0.201	0.769	0.484	0.529	0.660	1	0.340
DSP vs. MM		0.547	0.0002	0.005	0.012	0.563	0.01	1	0.662	0.106	0.011
DSG2 vs. MM		0.346	0.458	0.604	0.406	0.713	0.155	0.755	0.77	0.239	0.155
PKP2 vs. MM		1	0.62	0.911	0.728	0.479	0.305	0.309	1	0.104	0.476

**Table 5:** Comparison between SAECG and two-dimensional echo features at first and last evaluation in 53 desmosomal gene mutation carriers

Gene mutations	No.	Age of patients	RVOT (PLAX)/BSA	RVOT (PSAX)/BSA	RV FS	RVEDA, cm^2^	RV kinetic abn, n (%)	LVEDV, mL/m^2^	LVEF, %	LV kinetic abn, n (%)	Positive SAECG, n (%)
DSP	26	12 ± 3.9	12.72 ± 2.94	16.39 ± 3.54	37 ± 8	15 ± 4	3 (12)	56 ± 8	63 ± 5	1 (4)	3 (12)
DSP	26	19.6 ± 7	16.21 ± 4.48	18.25 ± 3.35	40 ± 9	22 ± 8	8 (31)	65 ± 14	59 ± 7	2 (8)	8 (31)
*P*			.001	.05	.209	.0002	.178	.006	.021	.546	.004
PKP2	12	13 ± 3.7	20.05 ± 4.72	23.11 ± 8.3	36 ± 7	21 ± 6	4 (33)	61 ± 9	62 ± 5	0 (0)	3 (40)
PKP2	12	20.8 ± 8.8	18.05 ± 5.91	19.16 ± 3.25	36 ± 7	23 ± 9	5 (42)	65 ± 11	62 ± 5	2 (17)	4 (33)
*P*			.369	.139	1	.528	.407	.340	1	.149	.184
DSG2	6	10.8 ± 3.6	18.32 ± 6.46	20.36 ± 6.72	37 ± 6	17 ± 6	1 (17)	58 ± 10	61 ± 3	0	1 (17)
DSG2	6	17.8 ± 5	17.15 ± 5.88	16.57 ± 9	40 ± 7	23 ± 10	1 (17)	58 ± 6	61 ± 3	0	1 (17)
*P*			.749	.420	.443	.300	1	1	1	1	1
MM	9	13 ± 4.9	21.71 ± 9.44	22.68 ± 9.15	39 ± 12	20 ± 7	5 (56)	56 ± 13	62 ± 8	2 (22)	5 (56%)
MM	9	26 ± 11.4	17.2 ± 4.28	23.06 ± 4.16	30 ± 6	23 ± 7	6 (67)	61 ± 11	50 ± 7	2 (22)	7 (78%)
*P*			.210	.911	.06	.376	.638	.391	.003	1	.335

**Table 6:** CMR findings in 21 desmosomal gene mutation carriers

	No. of patients (%)
Morphofunctional abnormalities	9 (43)
Isolated RV	5
Biventricular	4
LE on contrast enhanced CMR	12 (57)
RV isolated	2
RV inflow	0
RV inferior wall	1
RV outflow	0
RV anterior wall	1
RV apex	0
LV isolated	5
LV anterior wall	3
LV lateral wall	3
LV inferior wall	2
LV septum	2
Biventricular	5
RV inflow	1
RV inferior wall	3
RV outflow	0
RV anterior wall	4
RV apex	0
LV anterior wall	3
LV lateral wall	2
LV inferior wall	4
LV septum	1

## References

[1] Marcus F.I., Nava A., Thiene G. (2007). Arrhythmogenic right ventricular cardiomyopathy/dysplasia: Recent advances.

[2] Basso C., Corrado D., Marcus F.I., Nava A., Thiene G. (2009). Arrhythmogenic right ventricular cardiomyopathy. Lancet.

[3] McKoy G., Protonotarios N., Crosby A. (2000). Identification of a deletion in plakoglobin in arrhythmogenic right ventricular cardiomyopathy with palmoplantar keratoderma and woolly hair (Naxos disease). Lancet.

[4] Rampazzo A., Nava A., Malacrida S. (2002). Mutation in human desmoplakin domain binding to plakoglobin causes a dominant form of arrhythmogenic right ventricular cardiomyopathy. Am J Hum Genet.

[5] Alcalai R., Metzger S., Rosenheck S., Meiner V., Chajek-Shaul T. (2003). A recessive mutation in desmoplakin causes arrhythmogenic right ventricular dysplasia, skin disorder, and woolly hair. J Am Coll Cardiol.

[6] Gerull B., Heuser A., Wichter T. (2004). Mutations in the desmosomal protein plakophilin-2 are common in arrhythmogenic right ventricular cardiomyopathy. Nat Genet.

[7] Pilichou K., Nava A., Basso C. (2006). Mutations in desmoglein-2 gene are associated with arrhythmogenic right ventricular cardiomyopathy. Circulation.

[8] Syrris P., Ward D., Evans A. (2006). Arrhythmogenic right ventricular dysplasia/cardiomyopathy associated with mutations in the desmosomal gene desmocollin-2. Am J Hum Genet.

[9] Asimaki A., Syrris P., Wichter T. (2007). A novel dominant mutation in plakoglobin causes arrhythmogenic right ventricular cardiomyopathy. Am J Hum Genet.

[10] Bauce B., Basso C., Rampazzo A. (2005). Clinical profile of four families with arrhythmogenic right ventricular cardiomyopathy caused by dominant desmoplakin mutations. Eur Heart J.

[11] Dalal D., James C., Devanagondi R. (2005). Penetrance of mutations in plakophilin-2 among families with arrhythmogenic right ventricular dysplasia/cardiomyopathy. Circulation.

[12] Sen-Chowdhry S., Syrris P., Ward D., Asimaki A., Sevdalis E., McKenna W.J. (2007). Clinical and genetic characterization of families with arrhythmogenic right ventricular dysplasia/cardiomyopathy provides novel insights into patterns of disease expression. Circulation.

[13] van Tintelen J.P., Entius M.M., Bhuiyan Z.A. (2006). Plakophilin-2 mutations are the major determinant of familial arrhythmogenic right ventricular dysplasia/cardiomyopathy. Circulation.

[14] Protonotarios N., Tsatsopoulou A., Anastasakis A. (2001). Genotype-phenotype assessment in autosomal recessive arrhythmogenic right ventricular cardiomyopathy (Naxos disease) caused by a deletion in plakoglobin. J Am Coll Cardiol.

[15] den Haan A.D., Tan B.Y., Zikusoka M.N. (2009). Comprehensive desmosome mutation analysis in north Americans with arrhythmogenic right ventricular dysplasia/cardiomyopathy. Circ Cardiovasc Genet.

[16] Nava A., Bauce B., Basso C. (2000). Clinical profile and long term follow-up of 37 families with arrhythmogenic right ventricular cardiomyopathy. J Am Coll Cardiol.

[17] Daliento L., Turrini P., Nava A. (1995). Arrhythmogenic right ventricular cardiomyopathy in young versus adult patients: similarities and differences. J Am Coll Cardiol.

[18] Hamilton R.M., Fidler L. (2009). Right ventricular cardiomyopathy in the young: an emerging challenge. Heart Rhythm.

[19] Borry P., Evers-Kiebooms G., Cornel M.C. (2009). Genetic testing in asymptomatic minors: background considerations towards ESHG Recommendations. Eur J Hum Genet.

[20] McKenna W.J., Thiene G., Nava A. (1994). Diagnosis of arrhythmogenic right ventricular dysplasia/cardiomyopathy. Br Heart J.

[21] Marcus F.I., McKenna W.J., Sherrill D. (2010). Diagnosis of arrhythmogenic right ventricular cardiomyopathy/dysplasia (ARVC/D): Proposed modification of the task force criteria. Circulation.

[22] Nava A., Folino A.F., Bauce B. (2000). Signal averaged electrocardiogram in patients with arrhythmogenic right ventricular cardiomyopathy and ventricular arrhythmias. Eur Heart J.

[23] Kim R.J., Shah D.J., Judd R.M. (2003). How we perform delayed enhancement imaging. J Cardiovasc Magn Reson.

[24] Tandri H., Saranathan M., Rodriguez E.R. (2005). Noninvasive detection of myocardial fibrosis in arrhythmogenic right ventricular cardiomyopathy using delayed-enhancement magnetic resonance imaging. J Am Coll Cardiol.

[25] Cerqueira M.D., Weissman N.J., Dilsizian V. (2002). American Heart Association Writing Group on Myocardial Segmentation and Registration for Cardiac Imaging: Standardized myocardial segmentation and nomenclature for tomographic imaging of the heart: a statement for healthcare professionals from the Cardiac Imaging Committee of the Council on Clinical Cardiology of the American Heart Association. Circulation.

[26] Rampazzo A., Bauce B., Beffagna G. (2008). Prevalence of desmosomal protein mutations and clinical features in a large cohort of unrelated consecutive patients affected with arrhythmogenic right ventricular cardiomyopathy. Eur Heart J.

[27] Fressart V., Duthoit G., Donal E. (2010). Desmosomal gene analysis in arrhythmogenic right ventricular dysplasia/cardiomyopathy: spectrum of mutations and clinical impact in practice. Europace.

[28] Tan B.Y., Jain R., den Haan A.D. (2010). Shared desmosome gene findings in early and late onset arrhythmogenic right ventricular dysplasia/cardiomyopathy. J Cardiovasc Transl Res.

[29] Xu T., Yang Z., Vatta M. (2010). Compound and digenic heterozygosity contributes to arrhythmogenic right ventricular cardiomyopathy. J Am Coll Cardiol.

[30] Dalal D., Tandri H., Judge D.P. (2009). Morphologic variants of familial arrhythmogenic right ventricular dysplasia/cardiomyopathy: a genetics-magnetic resonance imaging correlation study. J Am Coll Cardiol.

[31] Dalal D., Molin L.H., Piccini J. (2006). Clinical features of arrhythmogenic right ventricular dysplasia/cardiomyopathy associated with mutations in plakophilin-2. Circulation.

[32] Gerull B., Heuser A., Wichter T. (2004). Mutations in the desmosomal protein plakophilin-2 are common in arrhythmogenic right ventricular cardiomyopathy. Nat Genet.

[33] Bauce B., Nava A., Beffagna G. (2010). Multiple mutations in desmosomal proteins encoding genes in arrhythmogenic right ventricular cardiomyopathy/dysplasia. Heart Rhythm.

[34] Yang Z., Bowles N.E., Scherer S.E. (2006). Desmosomal dysfunction due to mutations in desmoplakin causes arrhythmogenic right ventricular dysplasia/cardiomyopathy. Circ Res.

[35] Sen-Chowdhry S., Syrris P., Prasad S.K. (2008). Left-dominant arrhythmogenic cardiomyopathy: an under-recognized clinical entity. J Am Coll Cardiol.

[36] Basso C., Czarnowska E., Della Barbera M. (2006). Ultrastructural evidence of intercalated disc remodelling in arrhythmogenic right ventricular cardiomyopathy: an electron microscopy investigation on endomyocardial biopsies. Eur Heart J.

[37] Klauke B., Kossmann S., Gaertner A. (2010). De novo desmin-mutation N116S is associated with arrhythmogenic right ventricular cardiomyopathy. Hum Mol Genet.

[38] Syrris P., Ward D., Asimaki A. (2007). Desmoglein-2 mutations in arrhythmogenic right ventricular cardiomyopathy: a genotype-phenotype characterization of familial disease. Eur Heart J.

[39] Bhuiyan Z.A., Jongbloed J.D., van der Smagt J. (2009). Desmoglein-2 and desmocollin-2 mutations in Dutch arrhythmogenic right ventricular dysplasia/cardiomypathy patients: results from a multicenter study. Circ Cardiovasc Genet.

[40] De Bortoli M., Beffagna G., Bauce B. (2010). The p.A897KfsX4 frameshift variation in desmocollin-2 is not a causative mutation in arrhythmogenic right ventricular cardiomyopathy. Eur J Hum Genet.

[41] Thiene G., Nava A., Corrado D., Rossi L., Pennelli N. (1988). Right ventricular cardiomyopathy and sudden death in young people. N Eng J Med.

[42] Sen-Chowdhry S., Syrris P., Ward D., Asimaki A., Sevdalis E., McKenna W.J. (2007). Clinical and genetic characterization of families with arrhythmogenic right ventricular dysplasia/cardiomyopathy provides novel insights into patterns of disease expression. Circulation.

[43] Norman M., Simpson M., Mogensen J. (2005). Novel mutation in desmoplakin causes arrhythmogenic left ventricular cardiomyopathy. Circulation.

[44] Pilichou K., Remme C.A., Basso C. (2009). Myocyte necrosis underlies progressive myocardial dystrophy in mouse dsg2- related arrhythmogenic right ventricular cardiomyopathy. J Exp Med.

[45] Basso C., Corrado D., Thiene G. (2007). Arrhythmogenic right ventricular cardiomyopathy in athletes: diagnosis, management, and recommendations for sport activity. Cardiol Clin.

[46] Bauce B., Frigo G., Marcus F.I. (2008). Comparison of clinical features of arrhythmogenic right ventricular cardiomyopathy in males versus females. Am J Cardiol.

