# Supplementary material for: Clinical phenotype and diagnosis of arrhythmogenic right ventricular cardiomyopathy in pediatric patients carrying desmosomal gene mutations
Source: Heart Rhythm. 2011 Nov;8(11):1686–95. doi: 10.1016/j.hrthm.2011.06.026 (PMC3205183; doi:10.1016/j.hrthm.2011.06.026)
Supplement: Supplemental Table 1 [file mmc1.doc]

**Supplemental Table 1. Amino acid conservation and prediction of functional effect for detected missense mutations**

| **Gene** | **Exon** | **nucleotide change** | **AA change** | **AA conservation** | **Prediction of functional effect** | |
| --- | --- | --- | --- | --- | --- | --- |
|  |  |  |  |  | **SIFT** | **PolyPhen** |
| *PKP2* | Ex2 | c.227A>G | p.N76S | yes | Tolerated | Benign |
|  | Ex12 | c.2333T>C | p.I778T | yes | Affect protein function | Possibly damaging |
| *DSP* | Ex1 | c.88G>A | p.V30M* | no | Tolerated | Benign |
|  | Ex7 | c.897C>G | p.S299R^#^ | yes | Affect protein function | Possibly damaging |
|  | Ex9 | c.1124A>T | p.N375I | yes | Affect protein function | Probably damaging |
|  | Ex11 | c.1372A>T | p.N458Y | yes | Affect protein function | Probably damaging |
|  | Ex11 | c.1408A>G | p.K470E | yes | Affect protein function | Benign |
|  | Ex13 | c.1696G>A | p.A566T | yes | Affect protein function | Benign |
|  | Ex23 | c.3774C>A | p.D1258E | yes | Affect protein function | Benign |
|  | Ex23 | c.4803G>A | p.M1601I | yes | Affect protein function | Possibly damaging |
|  | Ex23 | c.4961T>C | p.L1654P | yes | Affect protein function | Possibly damaging |
|  | Ex23 | c.5324G>T | p.R1775I | yes | Affect protein function | Possibly damaging |
|  | Ex24 | c.7622G>A | p.R2541K | yes | Tolerated | Benign |
| *DSG2* | Ex4 | c.298G>C | p.G100R | yes | Affect protein function | Possibly damaging |
|  | Ex6 | c.689A>G | p.E230G | yes | Affect protein function | Probably damaging |
|  | Ex9 | c.1174G>A | p.V392I | yes | Tolerated | Benign |
|  | Ex13 | c.1912G>A | p.G638R | yes | Affect protein function | Probably damaging |
|  | Ex15 | c.2773C>T | p.P925S | yes | Tolerated | Probably damaging |

*In vitro and in vivo functional studies demonstrated a pathogenic role (ref. 34)

#Evidence of segregation with disease in a large family (ref. 4)
